# Parenting Educational Styles and Obesity Association in Mexican Children

**DOI:** 10.7759/cureus.65106

**Published:** 2024-07-22

**Authors:** Liliana Aurora Carrillo-Aguiar, Kenia Nahomi Ramos-Hinojosa, Ricardo Salas-Flores, Miriam Janet Cervantes-López, Orquídea Elizbeth Martínez-Pérez, Brian González-Pérez

**Affiliations:** 1 Education, Family Medicine Unit Number 77 of the Mexican Institute of Social Security (IMSS), Ciudad Madero, MEX; 2 Family Medicine, Family Medicine Unit Number 24 of the Mexican Institute of Social Security (IMSS), García, MEX; 3 School of Medicine of Tampico “Dr. Alberto Romo Caballero”, Universidad Autónoma de Tamaulipas (UAT), Tampico, MEX; 4 Family Medicine, Family Medicine Unit Number 77 of the Mexican Institute of Social Security (IMSS), Ciudad Madero, MEX; 5 Family Medicine, Family Medicine Unit Number 38 of the Mexican Institute of Social Security (IMSS), Tampico, MEX

**Keywords:** body mass index: bmi, educational level, overweight, parenting styles, child obesity

## Abstract

Introduction

Obesity can develop from childhood through adulthood and is influenced by genetics, family, and environmental factors. Parenting educational style is believed to contribute to an individual’s future weight status. This study aims to assess the connection between parenting educational style and weight-related issues.

Methods

The study involved 487 participants, including either the mother and/or father and their school-age child, aged 6-11, at a primary care unit in Mexico. Fifty-two records were excluded due to incomplete questionnaires, electronic records, and refusal of informed consent. The study group consisted of 435 adults and children who completed an adapted version of the Parenting Styles and Dimensions Questionnaire (PSDQ) tailored for the Mexican population. The researchers also gathered anthropometric measurements of the primary caregiver (parent) and the child from the electronic record to calculate their BMI and nutritional status. We used IBM SPSS Statistics for Windows, Version 25.0 (Released 2017; IBM Corp., Armonk, NY, USA) to analyze the data. The Pearson Chi-square and Fisher’s exact test were applied to examine interaction terms between variables, revealing a statistically significant p-value of <0.05.

Results

Out of the 435 patients examined, there were 229 (52.6%) children and 206 (47.3%) adult patients. Grade 2 obesity was present in 90 (39.3%) school-age children and 104 (50.5%) adult patients. The family’s parenting educational style, as determined by the PSDQ questionnaire, was found to be permissive in 143 (69.4%) patients, authoritarian in 33 (16.0%) patients, and authoritative in 30 (14.6%) patients.

Conclusions

Parenting educational style and the PSDQ tool can be used to assess how parents influence the development of obesogenic home environments. We observed that a permissive parenting educational style was linked to a more obesogenic environment, whereas an authoritative parenting educational style was linked to a less obesogenic environment.

## Introduction

Childhood obesity is a serious health issue associated with various problems, including metabolic syndrome, type 2 diabetes, sleep apnea, and low self-esteem. Obese individuals are more likely to remain obese into adulthood and experience related health issues [1]. In the US, about 17% of children are obese, leading to significant costs [2]. In Mexico, there is a concerning rise in childhood obesity [3]. In 2012, 9.7% of children under five were overweight or obese, and the national prevalence of overweight and obesity for school-age children was 34.4% [4]. Childhood obesity is influenced by various factors, such as family, neighborhoods, schools, the food industry, and culture [5].

The family environment has a significant impact on lifelong health habits and weight-related outcomes through different parenting educational styles. These styles, as defined by Baumrind, including authoritative, authoritarian, and permissive, play a vital role in childhood obesity by influencing eating behaviors and physical activity and serving as a clear intervention target [6]. Studies have shown links between parenting educational style and psychological factors affecting physical activity and eating behaviors. More research is needed to understand the relationship between school-age children’s BMI and physical activity [7]. This study aims to determine how different parenting educational styles, such as authoritative, authoritarian, or permissive, might impact weight-related outcomes and BMI in school-age children. Since BMI and parenting educational styles are factors that can be changed, this study is essential for creating evidence that can be used in intervention studies.

## Materials and methods

A cross-sectional and analytical study was conducted from January to December 2023; eligible patients (either the mother and/or father and their school-age child) between the ages of 6 and 11 were included from the Family Medicine Unit No. 77 of the Mexican Social Security Institute (IMSS), Torreón, Mexico. The sample size was 487 patients, calculated based on a 95% confidence level and a 5% margin of error, considering an infinite population size and factoring in a 10% loss or non-response rate. The sampling method was convenient, and mothers and their children who agreed to participate voluntarily were approached. Patients with certain endocrinological diseases (such as diabetes mellitus 1, hypothyroidism, and Cushing’s disease), cognitive/intellectual disabilities, or eating disorders were excluded. Before any procedures, authorization was obtained from the institutional ethics committee. The institutional review board approved this study under the number R-2022-2804-063.

The parenting educational styles variable was assessed using the Parenting Styles and Dimensions Questionnaire (PSDQ) [6]. The PSDQ comprises 62 Likert-type items and is designed to evaluate four global measures of parental styles based on the Baumrind typology: (1) authoritarian, (2) authoritative (democratic), (3) permissive (indulgent), and (4) neglectful. The questionnaire has shown sufficient reliability, with reported alphas ranging from 0.73 to 0.88, 0.82, and 0.88.

After explaining the study to adult patients, they were asked to sign an informed consent form attached to each questionnaire according to ethical principles for medical research. Once consent was obtained, we collected general information and surveyed the parental educational styles. Subsequently, the anthropometry of the parent (primary caregiver) was collected, and the child was taken from the electronic clinical record to calculate their BMI and nutritional status. We always ensured the confidentiality of the obtained data and the identity of the patients. Finally, we provided the test results to the parents or guardians.

We analyzed our data using IBM SPSS Statistics for Windows, Version 25.0 (Released 2017; IBM Corp., Armonk, NY, USA). To analyze the results, we used a binary logistic regression model. We recorded the sociodemographic characteristics of the sample using a capture sheet. Parenting educational styles were identified by classifying them using the median of each parenting style. Spearman’s Rho test (rs) was used after verifying the existence of non-normality in the data using the Kolmogorov-Smirnov test with Lilliefors correction. We used the Pearson Chi-square and Fisher’s exact test to examine interaction terms between variables. We considered a p-value <0.05 to be significant.

## Results

Out of the original 487 patients, 52 were excluded from the study: 31 due to incomplete questionnaire responses, 16 due to incomplete electronic records, and five due to refusal to accept informed consent. This group consists of 435 patients, including 229 (52.6%) children and 206 (47.3%) adult patients. Among them, 10.0% (N = 23) are part of single-parent families (single fathers or mothers). Among the child patients, 108 (47.2%) were female, and 121 (52.8%) were male. The average age of the children was 8 ± 1.62 years (range, 6-11 years) (Table 1).

**Table 1:** General characteristics of children

School-age child characteristics	N = 229 (%)
Sex, n (%)	
Female	108 (47.2)
Male	121 (52.8)
Age, years mean (SD)	8 (1.62)
Nutritional status, n (%)	
Normal	58 (25.3)
Overweight	35 (15.3)
Obese type 1	40 (17.5)
Obese type 2	90 (39.3)
Obese type 3	6 (2.6)
Single-parent family	23 (10.0)

The average age of the parents was 29 ± 2.64 years, ranging from 21 to 56 years. Among the patients, 79 (38.3%) had parents who completed their last university degree, 67 (32.5%) had finished primary school, and 13 (6.3%) had no schooling at all. Among the adult patients, 106 (89.5%) were diagnosed with obesity or overweight. Grade 2 obesity was the most common, present in 104 (50.5%) adult patients and 90 (39.3%) of their children. The family’s parenting educational style was classified based on the PSDQ questionnaire as permissive in 143 (69.4%) patients, authoritarian in 33 (16.0%) patients, and authoritative in 30 (14.6%) patients (Table 2).

**Table 2:** General characteristics of the parents

Parents characteristics	N = 206 (%)
Sex, n (%)	
Female	168 (81.6)
Male	38 (18.4)
Age, years mean (SD)	29 (2.64)
Educational level, n (%)	
University	79 (38.3)
Secondary school	47 (22.8)
Primary school	67 (32.5)
None	13 (6.3)
Nutritional status	
Normal	44 (21.4)
Overweight	21 (10.2)
Grade 1 obesity	17 (8.3)
Grade 2 obesity	104 (50.5)
Grade 3 obesity	20 (9.7)
Parenting educational style	
Authoritative	30 (14.6)
Authoritarian	33 (16.0)
Permissive	143 (69.4)

In the bivariate analysis, sex was not found to be associated with the presence of any of the parental educational styles (p > 0.05). However, most male school-age children were found to have authoritative and permissive styles, with 60% and 51.9%, respectively. Patients from single-parent families were found to have a permissive parental educational style, with 14 (9%) patients, although no statistically significant association was found (p > 0.05).

Families characterized by authoritative parenting educational styles were more likely to have completed university education, with 14 (40%) patients showing this trend (p = 0.012). On the other hand, the highest level of education of parents exhibiting an authoritarian parenting educational style was in primary school, with 16 (42.1%) of the patients falling into this category (p < 0.034). Additionally, the permissive parenting educational style was significantly associated with parents with secondary school educational levels (p = 0.045), found in 59 (37.8%) of the patients.

The nutritional status of patients was found to be linked to their parenting educational style. Patients with an authoritative parenting educational style were found to be associated with overweight (p = 0.025), while those with authoritarian parents were significantly associated with normal weight (p = 0.019). Notably, 62 (39.7%) patients with permissive parenting educational styles were found to have grade 2 obesity, indicating a significant association (p = 0.041) (Table 3).

**Table 3:** Correlation between PSDQ scores with general characteristics of child/parents PSDQ, Parenting Styles and Dimensions Questionnaire

Factor	Authoritative	p	Authoritarian	p	Permissive	p
Sex, n (%)		0.462		0.701		0.685
Female	14 (40)		19 (50)		75 (32.8)	
Male	21 (60)		19 (50)		81 (51.9)	
Single-parent family, n (%)	3 (8.6)	0.753	6 (15.8)	0.234	14 (9.0)	0.431
Educational level, n (%)						
University	14 (40.0)	0.012	14 (36.8)	0.085	37 (23.7)	0.853
Secondary school	10 (28.6)	0.062	7 (18.4)	0.102	59 (37.8)	0.045
Primary school	9 (25.7)	0.492	16 (42.1)	0.034	50 (32.1)	0.096
None	2 (5.7)	1	1 (2.6)	1	10 (6.4)	0.783
Nutritional status						
Normal	6 (17.1)	0.424	14 (36.8)	0.019	41 (26.3)	0.052
Overweight	14 (40.0)	0.025	9 (23.7)	0.631	19 (12.2)	0.411
Grade 1 obesity	7 (20.0)	1	4 (10.5)	0.922	29 (18.6)	0.62
Grade 2 obesity	7 (20.0)	1	11 (28.9)	0.395	62 (39.7)	0.041
Grade 3 obesity	1 (2.9)	0.924	0 (0.0)	0.881	5 (3.2)	0.112

## Discussion

This study’s findings, which offer a fresh perspective on the relationship between parenting educational styles and children’s weight status in Mexican American families, are significant. The results, in line with the parenting educational styles initially identified by Baumrind et al. [8]. and further developed by McCoby and Martin [9] in 1983, reveal that the authoritative parenting educational style associated with the parietal lobe was linked to a higher nutritional status (normal BMI) as a result of increased fruit consumption, physical activity, and a reduced risk of childhood obesity. In contrast, based on the results of this study, patients with an authoritative style were found to be associated with a normal nutritional status. These findings validate previous research and underscore the pivotal role of parenting educational styles in shaping children's health and well-being, providing a practical guide for parents and professionals in the field.

Several high-powered studies have found that, compared to authoritative parenting, authoritarian or negligent parenting is associated with an increased likelihood of obesity among preschool children but not among school-age children. This suggests that young children (age seven and below) are more vulnerable to obesity [10]. Several cross-sectional studies have indicated that there is no link between authoritarian parenting and higher BMI levels. Our research also noted a connection between normal BMI and authoritarian parenting but not with obesity. These studies involved children from various ethnic backgrounds, stressing the potential impact of cultural heritage and ethnicity on parental interactions, which warrants further investigation. Additionally, Fuemmeler et al. [11]. showed that parental authoritarian (β = -0.23, p < 0.05) and disengaged (β = -0.33, p < 0.05) parenting styles were linked to a slower linear increase in average BMI. Still, a less noticeable leveling off of BMI compared to the balanced parenting style.

The PSDQ was used to measure parenting educational styles, but it must be translated and adapted to ensure cultural sensitivity. Robinson et al. [12] found that the permissive style had fewer sensitive items and lower internal consistency compared to other styles. Similar trends were observed in validation studies of the PSDQ in Turkish, Portuguese, and Spanish [13-15]. The authoritative style includes warmth, involvement, reasoning, democratic participation, and well-intentioned/good treatment. The authoritarian style consists of verbal hostility, corporal punishment, a lack of reasoning/punitive strategies, and a lack of direction [16]. The permissive style is characterized by a lack of compliance, ignoring misbehavior, and a lack of self-confidence. The dimensions of parental authority based on the PSDQ items suggest a broader perspective [17]. A finer analysis may help identify differences based on the child’s behavior or issues and the parents’ characteristics.

This study obtained statistically significant data to support this finding. Previous research has shown a link between authoritative parenting and positive child well-being, including self-confidence and socioemotional adjustment, as well as the development of feeding practices that promote self-regulation and weight control [18-20]. Additionally, previous cross-cultural studies have indicated a relationship between parental styles, feeding practices, and self-regulation of food consumption, with the authoritative style being prevalent [20]. Carbajal and Ramírez [21]. conducted a study comparing parenting educational styles within each group (obese, normal weight) based on the sociocultural characteristics of the mother: partner status, occupation, and education. The study found that the parenting educational styles of mothers with obese children did not vary based on these sociocultural conditions. In contrast, our study found that education level is associated with authoritative and authoritarian parenting educational styles.

Barlow [22] recommends actively involving families in prevention efforts, promoting the use of authoritative parenting to support physical activity, discouraging weight-related outcomes and BMI, and discouraging the use of authoritarian parenting to control eating. Vollmer and Mobley [23] emphasize the need for more research to understand how parenting educational styles and feeding practices relate to obesity risk in children. Healthcare providers should identify parenting educational styles in children with weight issues within specific social and cultural contexts to tailor recommendations and interventions effectively.

It is crucial for future studies to include participants representing a more comprehensive range of social support conditions. Today, families are diverse, including nuclear, single-parent, and extended families. Extended families vary in terms of member permanence and kinship relationships with the mother. The results of this study have some limitations, as obtaining informed consent in clinical settings limits accessibility to cases, and not all referred mothers attend the Family Medicine Unit service, resulting in a double selection bias. To address these limitations, patient recruitment should be expanded, and study groups could be formed based on the age of the children to account for the developmental tasks of the children and their associated exercise styles and practices.

## Conclusions

The study findings illustrate how the parenting educational style and the PSDQ tool can be utilized to assess parental influence on the development of obesogenic home environments. The research revealed that a permissive parenting style was associated with a more obesogenic environment, while an authoritative parenting style correlated with being overweight and an authoritarian style with a normal BMI. These results underscore the significance of promoting children’s self-regulation of food intake through a parenting approach characterized by understanding the child’s needs, warmth, and involvement and employing reasoning and democratic participation for rule management.
